# The RNA-Binding Protein ProQ Promotes Antibiotic Persistence in Salmonella

**DOI:** 10.1128/mbio.02891-22

**Published:** 2022-11-21

**Authors:** Alisa Rizvanovic, Charlotte Michaux, Margherita Panza, Zeynep Iloglu, Sophie Helaine, E. Gerhart H. Wagner, Erik Holmqvist

**Affiliations:** a Department of Cell and Molecular Biology, Biomedical Centre, Uppsala University, Uppsala, Sweden; b Department of Microbiology, Centre for Molecular Microbiology and Infection, Imperial College London, London, United Kingdom; c Department of Microbiology, Harvard Medical School, Boston, Massachusetts, USA; National Institute of Child Health and Human Development (NICHD)

**Keywords:** ProQ, RNA-binding protein, persister formation, antibiotic persistence, *Salmonella*, flagella, antibiotic persisters, flagellar gene regulation

## Abstract

Bacterial populations can survive exposure to antibiotics through transient phenotypic and gene expression changes. These changes can be attributed to a small subpopulation of bacteria, giving rise to antibiotic persistence. Although this phenomenon has been known for decades, much remains to be learned about the mechanisms that drive persister formation. The RNA-binding protein ProQ has recently emerged as a global regulator of gene expression. Here, we show that ProQ impacts persister formation in Salmonella. *In vitro*, ProQ contributes to growth arrest in a subset of cells that are able to survive treatment at high concentrations of different antibiotics. The underlying mechanism for ProQ-dependent persister formation involves the activation of metabolically costly processes, including the flagellar pathway and the type III protein secretion system encoded on Salmonella pathogenicity island 2. Importantly, we show that the ProQ-dependent phenotype is relevant during macrophage infection and allows Salmonella to survive the combined action of host immune defenses and antibiotics. Together, our data highlight the importance of ProQ in Salmonella persistence and pathogenesis.

## INTRODUCTION

In nature, bacterial populations are constantly exposed to changing and stressful conditions and must rapidly adapt to survive. Phenotypic heterogeneity allows a bacterial population to split into subpopulations with different growth and survival properties as a result of changes in gene expression. Such heterogeneity underlies a phenomenon known as antibiotic persistence. Persister bacteria comprise a subpopulation that is transiently tolerant to antibiotics through growth arrest rather than genetic change (1–4). Persisters can resume growth, and if this occurs after antibiotic removal, a population of both persister and susceptible bacteria is reestablished (1–4). Increasing evidence suggests that the regrowth of persisters contributes to prolonged and recurrent infections (5–8) and facilitates the development of antibiotic resistance (9–11). For example, within macrophages, the intracellular pathogen Salmonella enterica serovar Typhimurium (Salmonella) forms a subpopulation of nongrowing persisters that can survive the combined action of host cell defense and antibiotics (2, 3, 8, 12) and promote the spread of antibiotic resistance plasmids upon regrowth (9).

Persisters may form spontaneously through fluctuations in gene expression. However, spontaneous persisters seem to be less common than triggered persisters, which are formed in response to multiple environmental signals such as nutrient limitation (13, 14), pH variation (2, 3), oxidative stress (15, 16), extracellular metabolites (17, 18), high cell density (19), and antibiotic exposure (20). However, the underlying mechanisms responsible for persister formation are not always understood, often because of ambiguous results that arise from difficulties in distinguishing antibiotic persistence from tolerance (21). Nevertheless, several mechanisms have repeatedly been shown to induce persister formation, including the activation of the stringent response (22–25) and the SOS response (26, 27), the induction of toxin-antitoxin modules (2, 6, 26–30), a drop in ATP levels (31–34), the induction of prophages (35, 36), and protein aggregation (37). Although persisters have generally been associated with cell dormancy, active cellular processes such as antioxidant scavenging and antibiotic efflux have been shown to promote persistence (13, 38). Moreover, intracellular Salmonella persisters actively secrete effector proteins into the host cell cytosol (12). Genome-wide screens have revealed many additional genes involved in prolonged growth arrest and persister formation (39–43). For example, the expression of virulence genes important for invasion imposes a metabolic burden on Salmonella, leading to the formation of a nongrowing antibiotic persister subpopulation (44, 45). Still, in most cases, the mechanistic role of genes involved in antibiotic persistence remains to be established.

The RNA-binding protein (RBP) ProQ has recently been recognized as a major posttranscriptional regulator of gene expression in Salmonella and Escherichia coli (46–50). ProQ binds to several hundred mRNAs and small RNAs (sRNAs) through the recognition of structured motifs (46, 47, 49–53). The regulatory outcomes of ProQ binding include stabilization of RNA targets (46–48, 52, 54) and translational mRNA repression via base-pairing sRNAs (48, 52). Through its RNA-binding and regulatory activities, ProQ contributes to several cellular responses in Salmonella and E. coli, such as adaptation to osmotic and chemical stress (55, 56), motility (46, 48, 57), biofilm formation (58), and bacterial virulence (48). Interestingly, several evolutionary experiments have identified adaptive loss-of-function mutations in *proQ*, suggesting that ProQ can negatively affect growth, which under certain conditions becomes disadvantageous (59–63). Consequently, we sought to determine whether and, if so, how ProQ affects Salmonella growth and what implications this has for bacterial persistence and pathogenesis.

In this study, we show that ProQ impacts the growth of Salmonella. Specifically, ProQ contributes to growth arrest in a subset of cells within a Salmonella population *in vitro* and, thus, the generation of distinct subpopulations of bacteria with different growth and survival properties. Accordingly, we show that ProQ promotes the formation of persisters that can survive high concentrations of different antibiotics, which are lethal to the rest of the population. In addition to providing an underlying mechanism for ProQ-dependent persister formation that relies on the expression of genes encoding flagella and the pathogenicity island 2 type III secretion system, we show that this phenotype is relevant during macrophage infection. Together, our data expand the physiological role of ProQ and show that this RBP is critical for the ability of Salmonella to survive antibiotic treatment.

## RESULTS

### ProQ reduces the growth rate of Salmonella.

**FIG 1:**
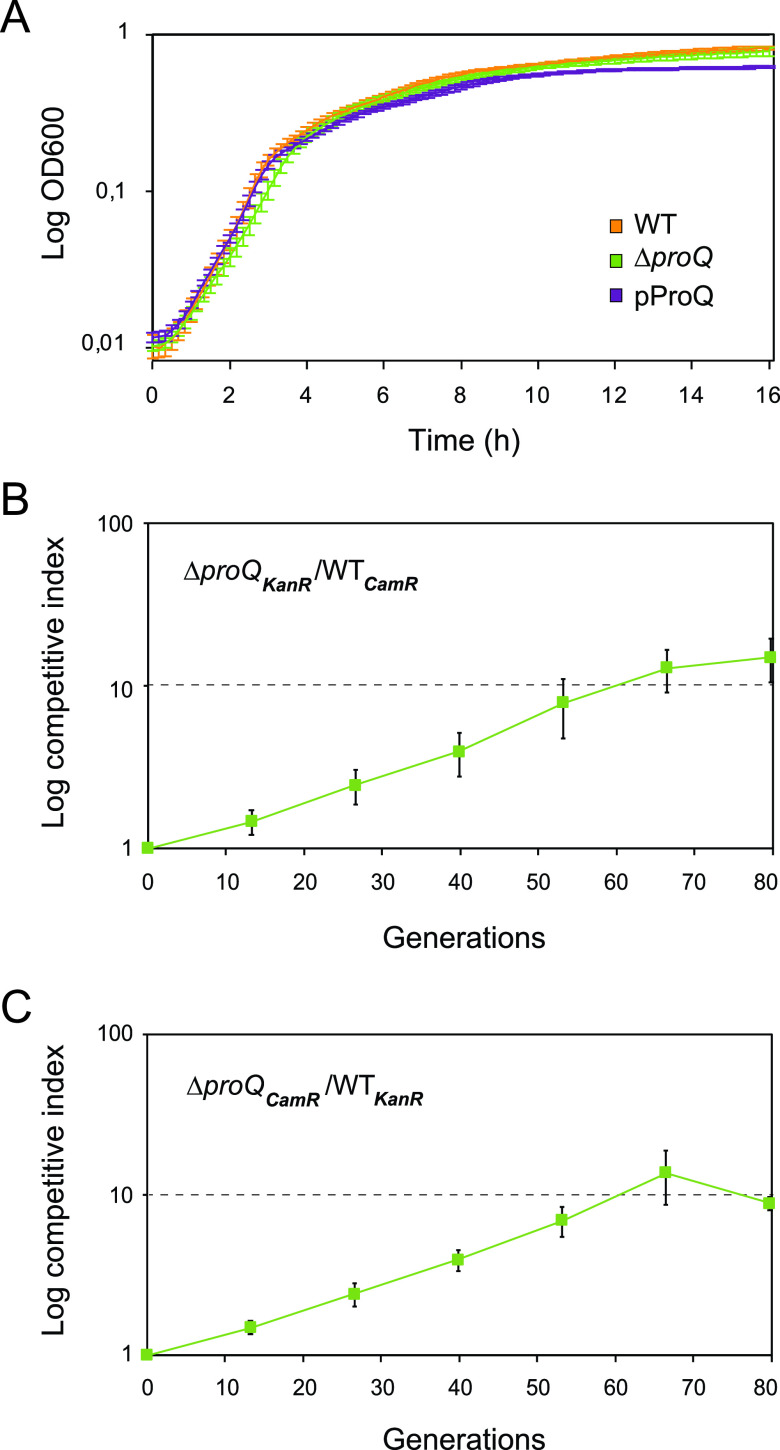
Effects of ProQ on Salmonella growth. (A) Growth curves of Salmonella SL1344 wild-type or Δ*proQ* cells carrying an empty control vector (WT, Δ*proQ*) or an IPTG-inducible *proQ* overexpression construct (pProQ). The optical density was monitored at 600 nm during growth in LB medium supplemented with IPTG (500 μM) in 96-well plates. The average values of six replicates with standard deviations (SD) are shown. (B and C) Growth competition experiments between Salmonella SL1344 strains. Salmonella wild-type and Δ*proQ* strains carrying antibiotic resistance marker genes (*CamR*, gene conferring chloramphenicol resistance; *KanR*, gene conferring kanamycin resistance) were mixed at a ratio of 1:1 in LB medium and incubated at 37°C. At roughly 24-h intervals, mixtures were plated on selective agar plates to determine CFU counts, and the remaining mixtures were serially passaged by 10,000-fold dilution in fresh LB medium for regrowth. Competitive indexes were calculated as the ratio of mutant cells to wild-type cells at the indicated generation divided by the initial ratio. Average values of six (generations 0 to 40) and three (generations 50 to 80) replicates with standard errors of the means (SEM) are shown.

Bacterial growth and survival depend on efficient adaptation to rapidly changing conditions. In Salmonella and E. coli, the RNA-binding protein ProQ plays a central role in adaptation by controlling the expression of a large number of genes (46–50). Previous studies demonstrated that adaptive mutations accumulate in the *proQ* gene in E. coli during laboratory evolution and confer a growth advantage over wild-type (WT) bacteria (59–61). These findings led us to investigate whether ProQ affects the growth of Salmonella. First, Salmonella SL1344 cells were grown in LB medium in 96-well plates for 16 h, and the optical density (OD) of cell populations was measured (Fig. 1A). The growth curves generated from the OD measurements suggested no obvious differences between the wild-type, Δ*proQ*, and ProQ complementation strains. We then asked whether a putative impact of ProQ on growth might be observable only during extended growth periods. To test this, competition experiments were performed. A Salmonella SL1344 Δ*proQ* strain carrying a chloramphenicol resistance marker gene was competed against a wild-type strain carrying a kanamycin resistance marker gene and vice versa. Cells from both strains were mixed in equal proportions and sequentially passaged in LB medium for 80 generations (six passages), during which CFU were determined every 13th generation. The competitive index (CI) was calculated as the ratio in CFU between Δ*proQ* and wild-type cells divided by the initial ratio at the start of the experiment. As seen in Fig. 1B and C, the Δ*proQ* strain outcompeted its wild-type counterpart irrespective of the antibiotic resistance marker used for selection. At the 80th generation, Δ*proQ* mutant cells outnumbered wild-type cells by approximately 10:1 (Fig. 1B and C). These results show that *proQ* imposes a burden on Salmonella growth at the population level.

### ProQ contributes to the formation of a growth-arrested Salmonella population.

**FIG 2:**
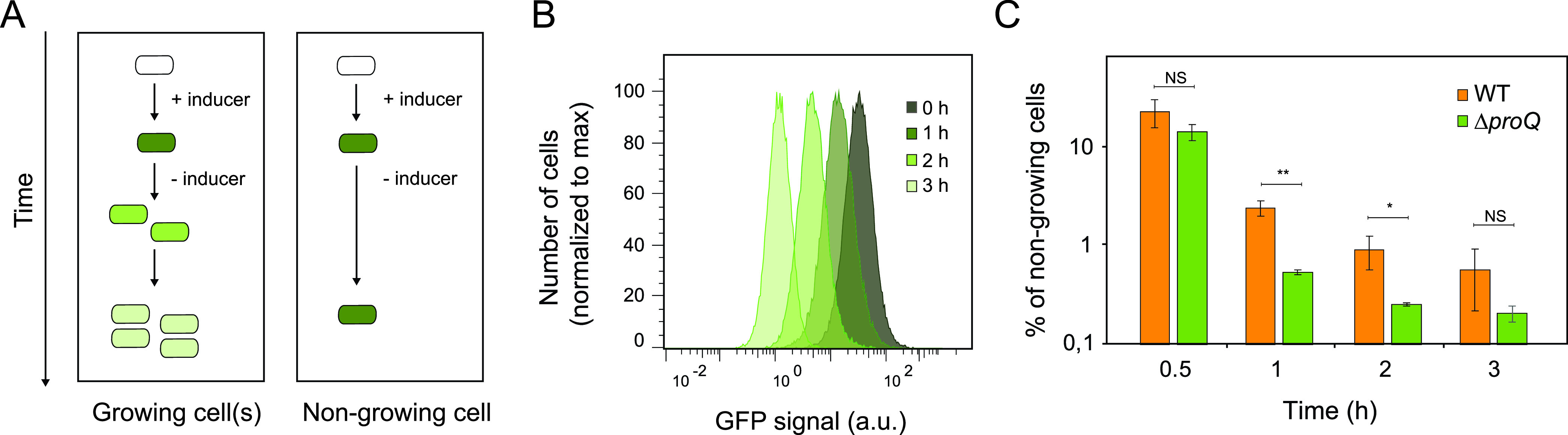
Single-cell analysis of Salmonella growth using fluorescence dilution *in vitro*. (A) Schematic of the fluorescence dilution method (2, 3, 12). Bacterial cells were labeled by inducing the expression of green fluorescence protein (GFP). After the accumulation of GFP, bacterial cells were transferred into fresh medium without an inducer, and changes in the GFP signal intensity were monitored by flow cytometry analysis. In growing cells, the GFP signal intensity decreases with each cell division. In nongrowing cells, the GFP signal intensity is retained. (B) Flow cytometry detection of green fluorescence in a Salmonella SL1344 wild-type population carrying plasmid pFCcGi (64). Bacterial cultures were grown to stationary phase in LB medium supplemented with arabinose (0.2%) to induce GFP expression. Upon regrowth in fresh LB medium without an inducer, dilution of the preformed pool of GFP was monitored during 0 h, 1 h, 2 h, and 3 h. Representative data are shown for one out of three replicates. Data for monitoring fluorescence dilution in SL1344 Δ*proQ* populations are shown in Fig. S1. a.u., arbitrary units. (C) Quantification of the percentage of nongrowing cells for Salmonella wild-type and Δ*proQ* strains (from panel B) 0.5 h, 1 h, 2 h, and 3 h after regrowth in fresh LB medium. The average values from three replicates with SD are shown. Statistical significance was determined using a two-tailed *t* test (*, *P* < 0.1; **, *P* < 0.01; NS, nonsignificant).

We next investigated the effect of ProQ on Salmonella growth at the single-cell level using fluorescence dilution (FD) (2, 3). This method is based on the dilution of a preformed pool of stable green fluorescence protein (GFP) after its induction has been stopped (Fig. 2A). In growing cells, the GFP signal intensity decreases 2-fold for each cell division, while nongrowing cells retain the initial high GFP intensity. For this purpose, the dual-fluorescence plasmid pFCcGi was used, which carries an arabinose-inducible GFP gene to monitor bacterial growth and a constitutively expressed mCherry gene for robust detection of bacterial cells during flow cytometry analysis (64). Salmonella SL1344 carrying pFCcGi was grown overnight in LB medium supplemented with arabinose to induce GFP expression. Cells were subsequently diluted 1,000-fold into fresh medium lacking the inducer and harvested at hourly intervals thereafter. Flow cytometry analysis revealed that the majority of Salmonella wild-type and Δ*proQ* cells had undergone division as observed by a decrease in GFP signal intensity over time (Fig. 2B; Fig. S1). Nevertheless, nongrowing cells that retained a high GFP signal were detected in both wild-type and Δ*proQ* populations (Fig. 2C). The Salmonella Δ*proQ* population displayed a significantly smaller fraction of nongrowing cells than the wild-type population during early exponential growth (Fig. 2C), indicating that ProQ contributes to the formation of growth-arrested cells within the Salmonella population.

10.1128/mbio.02891-22.1 FIG S1 Flow cytometry detection of green fluorescence in a Salmonella SL1344 Δ*proQ* population carrying the plasmid pFCcGi (64). Bacterial cultures were grown to stationary phase in LB medium supplemented with arabinose (0.2%) to induce GFP expression. Upon regrowth in fresh LB medium without an inducer, dilution of the preformed pool of GFP was monitored during 0 h, 1 h, 2 h, and 3 h. Representative data are shown for one out of three replicates. Data for monitoring fluorescence dilution in an SL1344 wild-type population are shown in Fig. 2B. Download FIG S1, EPS file, 1.4 MB. Copyright © 2022 Rizvanovic et al. 2022 Rizvanovic et al. https://creativecommons.org/licenses/by/4.0/ This content is distributed under the terms of the Creative Commons Attribution 4.0 International license.

### ProQ promotes Salmonella survival in the presence of antibiotics.

**FIG 3:**
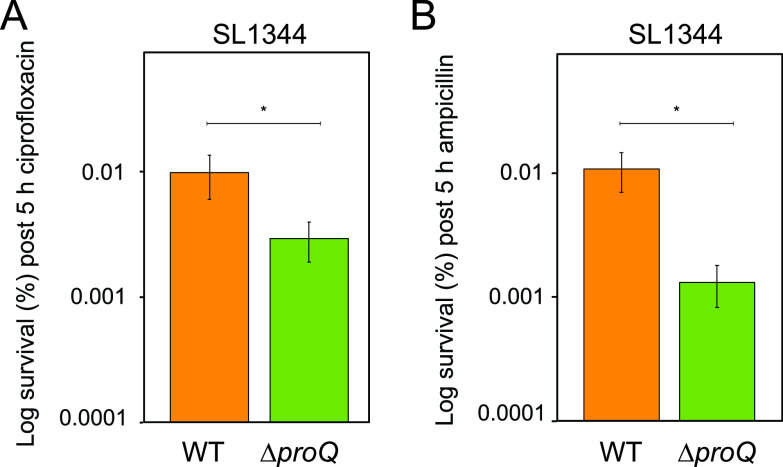
Effects of ProQ on Salmonella antibiotic persistence *in vitro*. Exponential-phase cultures of Salmonella SL1344 wild-type and Δ*proQ* strains were treated with ciprofloxacin (1 μg/mL; 60× MIC) (A) and ampicillin (50 μg/mL; 60× MIC) (B) for 5 h. CFU counts were determined before and after treatments to calculate the surviving fraction. Average values from 12 (A) and 6 (B) replicates with SEM are shown. Statistical significance was determined using a two-tailed *t* test (*, *P* < 0.1).

The formation of nongrowing cells within a bacterial population has been linked to antibiotic persistence (2–4, 65). We therefore assessed whether ProQ influences Salmonella antibiotic persistence. To this end, we performed persister assays with exponential-phase cultures treated with the DNA-damaging fluoroquinolone ciprofloxacin at 60× MIC for 5 h (Fig. 3A). Time-dependent killing curves revealed a biphasic pattern with a plateau of surviving persister bacteria (1) and showed that 5 h was an appropriate time point for determining persister levels under the tested conditions (Fig. S2). In these experiments, Salmonella deleted for *proQ* had three times lower persister levels than the wild-type strain. Given that persisters often exhibit a multidrug tolerance phenotype (66), persister assays were also carried out with the cell wall synthesis-inhibiting beta-lactam ampicillin, at 60× MIC for 5 h. Similar to the results presented in Fig. 3A, the Δ*proQ* strain had eight times lower persister levels than the wild-type strain (Fig. 3B). MIC tests confirmed that changes in survival were not due to changes in resistance to either ampicillin or ciprofloxacin (Table S1). Together, these results show that ProQ promotes persister formation in Salmonella.

10.1128/mbio.02891-22.2 FIG S2 Time-dependent antibiotic killing of the Salmonella wild-type strain. Exponential-phase cultures were treated with ciprofloxacin (1 μg/mL; 60× MIC) (A) or cefotaxime (100 μg/mL; 100× MIC) (B), and sample aliquots were taken at the indicated time intervals. CFU counts were determined before and after treatment and normalized to time zero. Average values from nine (A) or three (B) replicates and the standard errors of the means (SEM) are shown. Download FIG S2, EPS file, 1.4 MB. Copyright © 2022 Rizvanovic et al. 2022 Rizvanovic et al. https://creativecommons.org/licenses/by/4.0/ This content is distributed under the terms of the Creative Commons Attribution 4.0 International license.

10.1128/mbio.02891-22.3 TABLE S1 MICs determined for Salmonella strains using Etest strips. The average values from three replicates with SD are shown. Download Table S1, DOCX file, 0.01 MB. Copyright © 2022 Rizvanovic et al. 2022 Rizvanovic et al. https://creativecommons.org/licenses/by/4.0/ This content is distributed under the terms of the Creative Commons Attribution 4.0 International license.

### ProQ contributes to persister formation by increasing the expression of flagellar genes.

**FIG 4:**
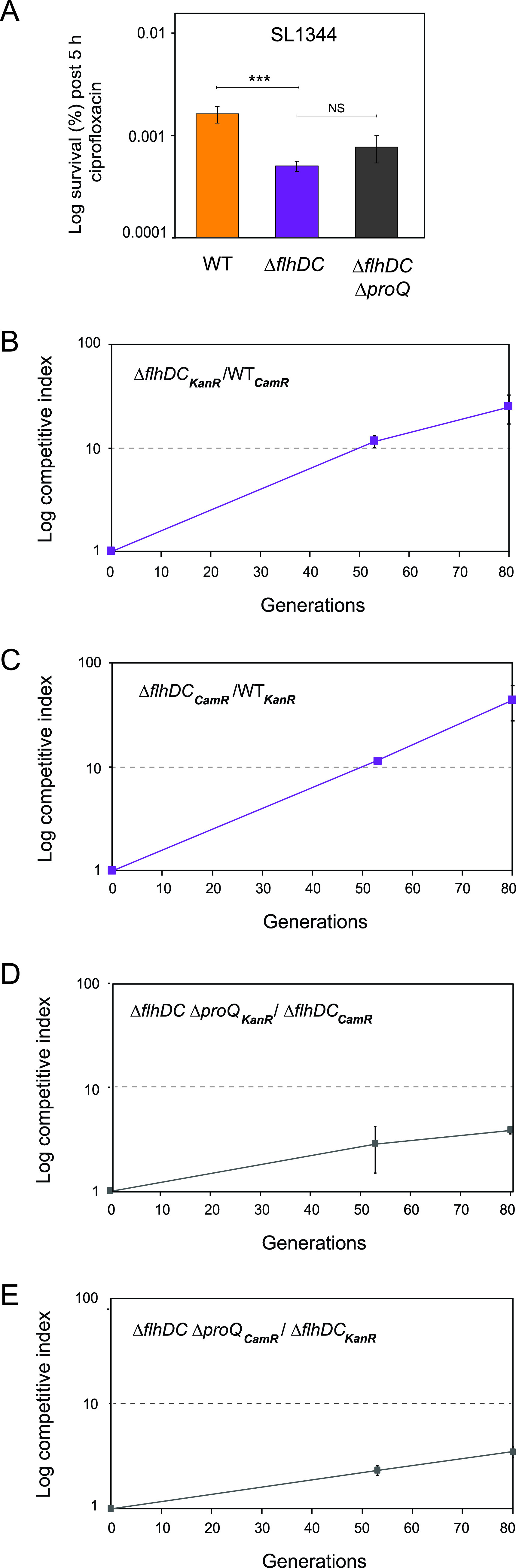
Effects of ProQ and FlhDC on antibiotic persistence *in vitro* and growth. (A) Exponential-phase cultures of Salmonella SL1344 wild-type, *ΔflhDC*, and Δ*flhDC* Δ*proQ* strains were treated with ciprofloxacin (1 μg/mL; 60× MIC) for 5 h. CFU counts were determined before and after treatment to calculate the surviving fraction. Average values from 5 (wild-type), 11 (Δ*flhDC*), and 8 (Δf*lhDC* Δ*proQ*) replicates with SEM are shown. Statistical significance was determined using a two-tailed *t* test (***, *P* < 0.001; NS, nonsignificant). (B to E) Growth competition experiments between Salmonella strains. Salmonella SL1344 wild-type and Δ*flhDC* strains (B and C) or Δ*flhDC* and Δ*flhDC* Δ*proQ* strains (D and E) carrying antibiotic resistance marker genes (*CamR*, gene conferring chloramphenicol resistance; *KanR*, gene conferring kanamycin resistance) were mixed at a ratio of 1:1 in LB medium and incubated at 37°C. At roughly 24-h intervals, mixtures were plated on selective agar plates to determine CFU counts, and the remaining mixtures were serially passaged by 10,000-fold dilution in fresh LB medium for regrowth. (B and C) Competitive indexes were calculated as the ratio of Δ*flhDC* cells to wild-type cells at the indicated generation divided by the initial ratio. (D and E) Competitive indexes were calculated as the ratio of Δ*flhDC ΔproQ* cells to Δ*flhDC* cells at the indicated generation divided by the initial ratio. The average values from three replicates with SD are shown.

We next asked by which mechanism ProQ promotes Salmonella persister formation. Recent transcriptomic analyses revealed that deletion of *proQ* leads to global effects on gene expression. For instance, genes located within the flagellar pathway are enriched among differentially expressed genes (46, 48). Previous studies using transposon mutagenesis in E. coli demonstrated that disruption of several flagellar genes decreases persister formation (42). Given that deletion of *proQ* in Salmonella reduces the expression of most flagellar genes (46–48, 57), we asked whether flagellar synthesis has a role in the formation of ProQ-dependent persister cells. To this end, we constructed a Salmonella SL1344 strain lacking *flhDC*, encoding the master regulator that controls the expression of all flagellar genes (67, 68). Deletion of *flhDC* resulted in a significant reduction in persister levels compared to the wild-type strain following 5 h of ciprofloxacin treatment (Fig. 4A), indicating that flagellar synthesis contributes to persister formation in Salmonella. Strikingly, deleting *proQ* did not reduce the persister levels in the Δ*flhDC* strain (Fig. 4A). This shows that ProQ-dependent effects on persister frequencies require a functional flagellar pathway and indicates that ProQ-dependent persister formation in Salmonella is linked to ProQ-dependent regulation of flagellar gene expression (57).

The buildup and operation of flagella is estimated to use 5 to 10% of the total cell energy budget (69, 70) and, hence, confers a substantial cost for Salmonella growth. It is therefore possible that the decreased persister levels in the Δ*flhDC* mutant (Fig. 4A) are linked to the increased availability of energy resources otherwise needed for producing flagella. To test this, we competed a Salmonella SL1344 Δ*flhDC* strain carrying a chloramphenicol resistance marker gene against a wild-type strain carrying a kanamycin resistance marker gene and vice versa (Fig. 4B and C). As expected, strains lacking *flhDC* strongly outcompeted the wild-type strain by 25:1 at the 80th generation, verifying that flagellar gene expression confers a growth disadvantage for Salmonella.

Since ProQ-dependent persister formation requires a functional flagellar pathway (Fig. 4A), we next asked whether the observed growth disadvantage conferred by ProQ (Fig. 1B and C) could be attributable to the expression of flagellar genes. To address this, the competitive fitness of ProQ was studied in a Δ*flhDC* background. The Salmonella SL1344 Δ*proQ ΔflhDC* strain carrying a chloramphenicol resistance marker gene was competed against a Δ*flhDC* strain carrying a kanamycin resistance marker gene and vice versa (Fig. 4D and E). In contrast to previous results (Fig. 1B and C), the growth advantage conferred by the deletion of *proQ* was strongly reduced in the Δ*flhDC* background (Fig. 4D and E). Together, these results suggest that deletion of *proQ*, which entails reduced activation of flagellar synthesis (46, 48, 57), leads to a growth advantage in Salmonella.

### ProQ contributes to persister formation by increasing the expression of genes encoding the SPI-2 type III secretion system.

**FIG 5:**
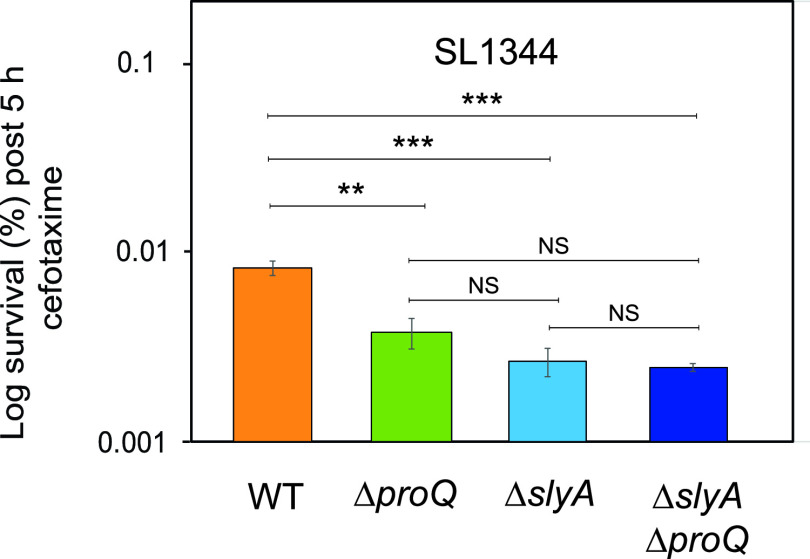
Effects of ProQ and SlyA on antibiotic persistence *in vitro.* Exponential-phase cultures of Salmonella SL1344 wild-type, Δ*proQ*, Δ*slyA*, and Δ*slyA* Δ*proQ* strains in acidic SPI-2 medium were treated with cefotaxime (100 μg/mL) for 5 h. CFU counts were determined before and after treatment to calculate the surviving fraction. The average values from four replicates with SEM are shown. Statistical significance was determined using a two-tailed *t* test (***, *P* < 0.001; **, *P* < 0.01; NS, nonsignificant).

Activation of virulence-related processes in a nonhost environment also confers a metabolic cost for Salmonella, and the resulting growth reduction has been linked to antibiotic persistence (44, 45). The data in Fig. 4 suggest that ProQ promotes the formation of persisters *in vitro* through the activation of a costly process, the flagellar pathway. We asked whether the activation of other metabolically costly processes by ProQ promotes persister formation. Recent transcriptomic analyses showed that deletion of *proQ* leads to a downregulation of genes in Salmonella pathogenicity island 2 (SPI-2), which encode a type III secretion system (T3SS) important for intracellular survival (48). To test whether ProQ affects persister formation through the SPI-2-encoded T3SS, we constructed a Salmonella SL1344 strain lacking *slyA*, which encodes one of the main transcriptional activators of the SPI-2 locus (71). We performed persister assays in acidic SPI-2-inducing medium with exponential-phase cultures treated with the beta-lactam cefotaxime for 5 h (Fig. 5). Consistent with previous results (Fig. 3 and 4A), Salmonella deleted for *proQ* had two times lower persister levels than the wild-type strain. Furthermore, the deletion of *slyA* resulted in a significant reduction in persister levels compared to the wild-type strain, indicating that the production of the SPI-2 T3SS contributes to the antibiotic persistence of Salmonella. Notably, deleting *proQ* did not reduce the persister levels in the *slyA* mutant. Thus, ProQ activation of the SPI-2-encoded T3SS (48) promotes Salmonella survival in the presence of antibiotics under *in vitro* conditions that mimic the intracellular environment.

### ProQ promotes Salmonella survival in the presence of antibiotics during macrophage infection.

**FIG 6:**
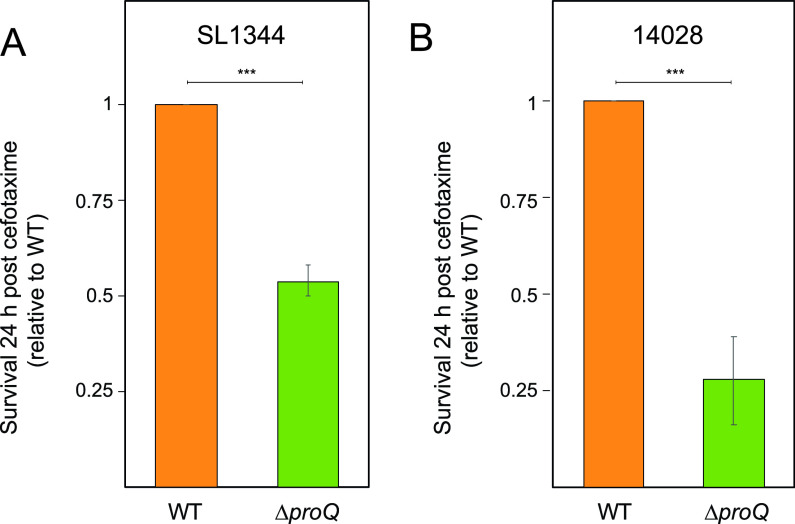
Effects of ProQ on Salmonella antibiotic persistence during macrophage infection. Mouse bone marrow-derived macrophages were infected with wild-type or Δ*proQ* strains of Salmonella SL1344 (A) and 14028 (B) and treated with cefotaxime (100 μg/mL; 100× MIC) for 24 h. For Salmonella SL1344, the infection medium was supplemented with 2 mM histidine. Intracellular Salmonella cells were released from macrophages, and the surviving fraction was determined by CFU counts and normalized to wild-type levels. Average values from six replicates (A) and four replicates (B) with SEM are shown. Statistical significance was determined using a two-tailed *t* test (***, *P* < 0.001).

Salmonella is an intracellular pathogen that produces high levels of persister cells following internalization by macrophages (2, 3, 8, 12). As our *in vitro* work revealed that ProQ contributes to persister formation in Salmonella (Fig. 4 and 5), we asked whether this phenotypic effect could be observed *in vivo*. Mouse bone marrow-derived macrophages (BMDMs) were infected with Salmonella and treated with the cell wall synthesis-inhibiting beta-lactam cefotaxime at 100× MIC for 24 h. The fraction of surviving cells was determined by release from macrophages and CFU counts. Consistent with our *in vitro* results (Fig. 3 and 5A), the number of intramacrophage persister cells decreased by 50% in Salmonella SL1344 lacking *proQ* compared to the wild-type strain (Fig. 6A; Table S5). Given that the strain SL1344 is auxotrophic for histidine (72), we reasoned that intracellular limitation of this amino acid could in itself affect Salmonella growth and, thus, mask the persister population effect (73). Therefore, persister assays were repeated with Δ*proQ* and wild-type strains of Salmonella 14028, which are proficient in histidine biosynthesis (74, 75). The number of intramacrophage persister cells decreased by approximately 75% in 14028 Δ*proQ* compared to the wild-type strain (Fig. 6B). The MIC for cefotaxime was not affected by the *proQ* deletion, neither in SL1344 nor in 14028, ruling out antibiotic resistance as a plausible explanation for differences in persister levels (Table S1). Together, this shows that ProQ promotes Salmonella persisters during macrophage infection.

10.1128/mbio.02891-22.7 TABLE S5 CFU counts from a representative *in vivo* persister assay. Download Table S5, DOCX file, 0.01 MB. Copyright © 2022 Rizvanovic et al. 2022 Rizvanovic et al. https://creativecommons.org/licenses/by/4.0/ This content is distributed under the terms of the Creative Commons Attribution 4.0 International license.

**FIG 7:**
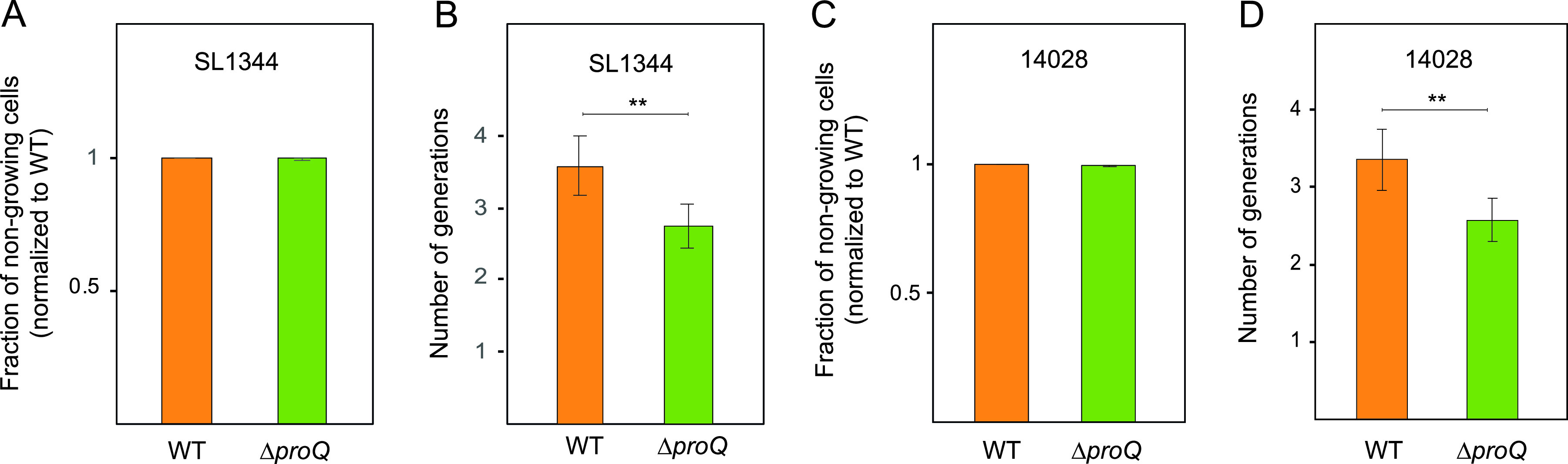
Single-cell analysis of Salmonella growth using fluorescence dilution during macrophage infection. Salmonella SL1344 and 14028 wild-type and Δ*proQ* strains were grown to stationary phase in LB medium supplemented with arabinose (0.2%) to induce GFP expression. Mouse bone marrow-derived macrophages were infected with the preinduced Salmonella cells for 16 h. Intracellular Salmonella cells were released from macrophages and subjected to flow cytometry analysis. (A and C) Quantification of the fraction of nongrowing cells. (B and D) Quantification of the number of undergone generations. The average values from six replicates with SD are shown. Statistical significance was determined using a two-tailed *t* test (**, *P* < 0.01).

The effect of ProQ on the survival of intracellular Salmonella in the presence of antibiotics (Fig. 5) might reflect effects on bacterial growth. To test if ProQ affects single-cell growth within the intracellular Salmonella population, the FD method was used (Fig. 2A). Mouse bone marrow-derived macrophages were infected with preinduced Salmonella carrying the dual-fluorescence plasmid pFCcGi. After 16 h of infection, the released Salmonella cells were subjected to flow cytometry for quantification of growing and nongrowing fractions (Fig. 7). Interestingly, no obvious differences in the fraction of nongrowing cells were observed between SL1344 wild-type and Δ*proQ* intracellular populations (Fig. 7A). This contrasted with the smaller fraction of nongrowing cells observed for the Δ*proQ* population that formed in the LB medium (Fig. 2). Quantification of proliferation by FD revealed significantly slower growth for single cells within the Δ*proQ* population than in the wild-type population (Fig. 7B). Similar results were observed for mouse bone marrow-derived macrophages infected with preinduced Salmonella 14028 carrying pFCcGi. The 14028 wild-type and Δ*proQ* populations showed no differences in the fraction of nongrowing cells, but single cells within the Δ*proQ* population grew to a lesser extent than the wild-type cells (Fig. 7C and D). Together, these results indicate that ProQ promotes the growth (Fig. 7) and survival (Fig. 6) of single cells within a Salmonella population during macrophage infection, presumably through the activation of the SPI-2-encoded T3SS (48), which in contrast to flagella, is upregulated during intracellular growth (76) and is required for the survival of antibiotic persister cells (Fig. 5) (12).

## DISCUSSION

Bacteria produce phenotypic subpopulations of nongrowing persisters that can survive exposure to antibiotics (77, 78). Understanding the molecular pathways essential for the formation of these persisters may lead to new strategies for their elimination. To date, several global regulators, toxin-antitoxin modules, and metabolic enzymes have been linked to persister formation (79–81). Here, we show for the first time that the global RNA-binding protein ProQ promotes persister formation.

**FIG 8:**
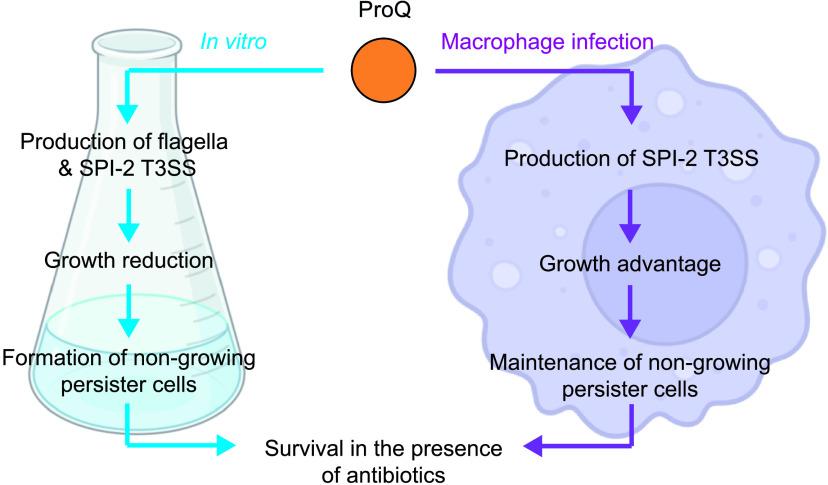
Schematic model of ProQ-dependent persister formation. During the growth of Salmonella monocultures under standard laboratory conditions, ProQ promotes the expression of energetically costly but dispensable processes such as flagella and the SPI-2 T3SS. This leads to decreased growth, an increased frequency of nongrowing cells, and higher persister levels. When Salmonella resides inside macrophages, ProQ promotes the expression of the SPI-2 T3SS, which is indispensable for the survival, growth, and maintenance of persisters under this condition.

In Fig. 1, we show that ProQ impacts the growth of Salmonella. Specifically, ProQ contributes to the formation of nongrowing cells within the Salmonella population (Fig. 2) that are able to survive treatment at high concentrations of antibiotics (Fig. 3) without changes in resistance (Table S1) and, thus, represent persisters. ProQ promotes the formation of these nongrowing persisters in laboratory medium by activating flagellar synthesis (Fig. 4) and the SPI-2-encoded T3SS (Fig. 5). We reveal that the ProQ-dependent impact on persisters is relevant both in bacterial monoculture and during host cell infection (Fig. 8).

Persisters may be generated through stochastic fluctuations in gene expression or, more commonly, upon perceiving a stress signal (2, 3, 14, 17, 19, 20). Multiple stress signals have been shown to trigger persister formation, the most common one being starvation (13), although active cellular processes also can promote persistence (reviewed in reference 80). The *in vitro* persister assays used in this study (Fig. 3 to 5) were performed by diluting a starved overnight culture into fresh medium. The culture was incubated until it reached exponential phase, and antibiotics were added to score the persister cells. It is therefore possible that the ProQ-induced persisters (Fig. 3 to 5) were triggered already by the preceding starvation phase. In line with this, we observed a decline in nongrowing cells after diluting a stationary-phase culture into fresh growth medium and a decrease in the fraction of nongrowing cells upon deletion of *proQ* (Fig. 2). Although outside the scope of this study, it would be interesting to monitor ProQ’s effect on the formation of nongrowing cells and persisters upon entry into stationary phase. ProQ-dependent persister formation was also observed during macrophage infection (Fig. 6). After uptake by macrophages, Salmonella encounters an acidified and nutrient-limited environment, which has been linked to persister formation (2). It is therefore possible that the ProQ-induced persisters in macrophages (Fig. 6) were triggered by starvation as well.

Previous work has suggested the role of ProQ in the regulation of flagellar genes (46–48, 57). We show here that ProQ promotes persister formation by activating flagellar synthesis (Fig. 4). Thus, it appears that ProQ impacts not only flagellar synthesis itself but also persister formation through the flagellar pathway. Work in E. coli has shown that flagellar synthesis contributes to the formation of persisters, but the underlying mechanism is not completely known (42). Our work suggests that flagellar synthesis confers a growth disadvantage for bacterial cells (Fig. 4) and, thereby, entails increased persistence. A similar phenomenon has been observed in Pseudomonas aeruginosa, where a low-persister strain outcompeted a high-persister strain in both the absence and presence of antibiotics (82). In relation to this, it is tempting to speculate that the *proQ* deletion strain would lose its competitive advantage in the presence of antibiotics. After uptake by macrophages, flagella are no longer required for movement, and Salmonella strongly downregulates flagellar synthesis (83, 84). Therefore, it is likely that ProQ promotes persistence *in vivo* (Fig. 6) through a mechanism other than the activation of the flagellar pathway.

Inside macrophages, Salmonella induces the expression of a T3SS encoded on its pathogenicity island 2 and uses it to secrete effector molecules that interfere with host cell defenses (85, 86). Recently, it was shown that the survival, but not formation, of Salmonella persisters within macrophages is dependent on a functional SPI-2-encoded T3SS that allows reprogramming of the host (12). ProQ positively regulates the expression of SPI-2 genes; in its absence, the majority of these genes are downregulated (48). We show here that a functional SPI-2 T3SS is required for ProQ-dependent persister formation during *in vitro* conditions that mimic the host cell environment (Fig. 5). We therefore speculate that ProQ also promotes the survival of Salmonella persisters within macrophages (Fig. 6) through the activation of the SPI-2 T3SS. Interestingly, ProQ does not seem to affect the size of the nongrowing subpopulation in macrophages (Fig. 7A, C), in agreement with the fact that SPI-2 promotes persister survival but not formation. Indeed, if all intracellular persisters stem from the nongrowing population, not all nongrowers are persisters (2), and it requires additional factors other than growth arrest to successfully survive the combined action of antibiotics and macrophages (2, 12). It thus appears that ProQ is involved in the maintenance of intramacrophage persister cells rather than their formation. Like SPI-2, ProQ promotes the growth rate of the growing intracellular Salmonella population (Fig. 7B and D) (3).

While ProQ seems to promote the growth of Salmonella during macrophage infection (Fig. 7B to D), it reduces bacterial growth under standard laboratory conditions (Fig. 1B and C). We speculate that ProQ-dependent activation of flagella and SPI-2 genes (46, 48) in nonhost environments confers a metabolic cost for bacterial cells, which reduces bacterial growth (Fig. 4B to E) and increases the fraction of persister cells (Fig. 4A and 6). In line with this, the activation of other virulence-related processes, such as the SPI-1-encoded T3SS that is important for host cell invasion, has been shown to reduce Salmonella growth in nonhost environments and lead to the formation of an antibiotic persister subpopulation (44, 45). Thus, the activation of metabolically costly processes may be a more general mechanism by which Salmonella persister populations can form and evade antibiotics and is, in part, dependent on ProQ.

Previous work on Salmonella has reported that ProQ contributes to several cellular processes important for pathogenesis, such as motility (46, 48), biofilm formation (58), and bacterial virulence (48). We extend this view by demonstrating that ProQ contributes to the survival of Salmonella to antibiotic exposure. Taken together, our work expands the role of ProQ and highlights the importance of this RBP for Salmonella pathogenesis.

## MATERIALS AND METHODS

### Strains and growth conditions.

All Salmonella strains used in this study are listed in Table S2. Salmonella cells were grown aerobically at 37°C with shaking at 220 rpm in standard Luria-Bertani broth (LB) medium or SPI-2 medium (87). If applicable, the following antibiotics were added to the growth medium: kanamycin (50 μg/mL), ampicillin (100 μg/mL), tetracycline (15 μg/mL), and chloramphenicol (30 μg/mL).

10.1128/mbio.02891-22.4 TABLE S2 Strains used in this study. Download Table S2, DOCX file, 0.02 MB. Copyright © 2022 Rizvanovic et al. 2022 Rizvanovic et al. https://creativecommons.org/licenses/by/4.0/ This content is distributed under the terms of the Creative Commons Attribution 4.0 International license.

### Plasmid construction.

Plasmids used in this study are listed in Table S3. The construction of plasmids pAR007, pAR009, and pFCcGi has been described elsewhere (57, 64).

10.1128/mbio.02891-22.5 TABLE S3 Plasmids used in this study. Download Table S3, DOCX file, 0.02 MB. Copyright © 2022 Rizvanovic et al. 2022 Rizvanovic et al. https://creativecommons.org/licenses/by/4.0/ This content is distributed under the terms of the Creative Commons Attribution 4.0 International license.

### Strain construction.

Oligonucleotides used in this study are listed in Table S4. The Δ*flhDC* mutant strain (EHS-2093) was constructed by lambda Red recombination (88) using plasmid pKD4 (89). First, a kanamycin resistance gene was amplified (EHO-1118/1119) from strain JVS-11364 (46), inserted into a Salmonella wild-type strain, and subsequently transferred to a Salmonella wild-type strain by P22 transduction (88). Second, the antibiotic resistance cassette was removed using plasmid pCP20 (90). The Δ*slyA* mutant strain (EHS-1880) was constructed by P22 transduction. First, the *slyA* gene was deleted from JVS-1574 by using phage lysate from strain 1867 from the McClelland collection (91). Second, the antibiotic resistance cassette was removed using plasmid pCP20. The *proQ* gene was deleted from EHS-1880, EHS-2391, EHS-2392, EHS-2093, and EHS-2209 by using P22 phage lysate from JVS-11364. The antibiotic resistance cassettes were removed using plasmid pCP20. The STM1553 pseudogene was deleted from strains JVS-1574, EHS-1880, EHS-1882, EHS-2093, and EHS-2154 by use of P22 phage lysates from SK3313 (92) and SK3318 (92).

10.1128/mbio.02891-22.6 TABLE S4 Oligonucleotides used in this study. Download Table S4, DOCX file, 0.01 MB. Copyright © 2022 Rizvanovic et al. 2022 Rizvanovic et al. https://creativecommons.org/licenses/by/4.0/ This content is distributed under the terms of the Creative Commons Attribution 4.0 International license.

### Optical density measurements.

Overnight cultures were diluted 1:1,000 into fresh LB medium supplemented with 500 μM isopropyl-β-d-thiogalactoside (IPTG; Sigma). Cultures were grown in 96-well plates (Costar) at 37°C, and the optical density at 600 nm (OD_600_) was measured every 10 min for 16 h in a plate reader (Tecan Infinite Pro). The background was removed by subtracting the OD values obtained from wells with LB medium from those of wells with bacterial cells. Mean values and standard deviations (SD) were calculated for every biological replicate.

### Competition experiments.

Overnight cultures of tagged mutant and parental strains were mixed in a 1:1 ratio. At roughly 24-h intervals, cell mixtures were serially passaged for 6 days by 10,000-fold dilution into 1 mL fresh LB medium or SPI-2 medium and incubated at 37°C with shaking at 220 rpm. On the indicated days, the ratio of mutant to parent was measured by CFU counts on selective agar plates. Competitive indexes were calculated as the ratio of the CFU of mutant to parental cells divided by the initial ratio.

### Fluorescence dilution method *in vitro*.

Single colonies of Salmonella strains carrying the plasmid pFCcGi were inoculated in LB medium supplemented with 0.2% l-arabinose (Sigma) and incubated overnight. The overnight cultures were diluted to an OD of 0.001 in fresh LB medium in the absence of an inducer and grown for 3 h. At hourly intervals, bacterial pellets were resuspended in sterile phosphate-buffered saline (PBS) and loaded in a 96-well polystyrene plate for single-cell analysis of GFP and mCherry fluorescence. Flow cytometry analysis was performed using a MACSQuant VYB flow cytometer (Miltenyi Biotec). GFP was excited with a blue laser (488 nm; band-pass filter, 525/50 nm; channel B1). mCherry was excited with a yellow laser (561 nm; band-pass filter, 615/20 nm; channel Y2). A total of 100,000 events were recorded for each sample. Data were acquired with the MACSQuantify software (Miltenyi Biotec) and processed with FlowJo software (Beckton, Dickinson, and Company). For the identification of bacterial cells, the gate was set based on the constitutive mCherry signal. For the characterization of nongrowing cells, the gate was set on a retained GFP signal. The percentage of growing bacteria was calculated using the equation x = 1/1 + 2^*n*^ × (*nr*)/*r*, where *x* is the percentage of growing bacteria, *n* is the number of generations that growing bacteria undergo, *nr* is the fraction of nongrowing bacteria measured by the retained GFP signal, and *r* is the fraction of growing bacteria measured by dilution in GFP signal. The number of generations was determined by calculating the log_2_ of the ratio in the geometric mean of GFP between the initial nondividing population and the dividing population.

### *In vitro* persister assays.

Overnight cultures were diluted to an OD_600_ of 0.02 in fresh LB medium or an OD_600_ of 0.04 in fresh SPI-2 medium and incubated at 37°C with shaking at 220 rpm until reaching exponential phase (OD_600_ of 0.25 to 0.35). At that point, ciprofloxacin (1 μg/mL; 60× MIC), ampicillin (50 μg/mL; 60× MIC), or cefotaxime (100 μg/mL; 100× MIC) was added to cultures. Before and 5 h after antibiotic treatment, bacterial pellets were washed once and diluted in PBS. Samples were plated on agar plates and incubated at 37°C for 16 h. CFU counts were determined from plates containing 30 to 300 bacterial colonies. The surviving fraction of cells was determined by the ratio of CFU between treated and untreated samples.

### Cell culture and infection of macrophages.

Bone marrow-derived macrophages (BMDMs) were extracted from the tibia and femur of C57BL/6 female mice (Charles River) according to a UK Home Office Project License and grown as described previously (12). For infection, bacteria from overnight cultures were opsonized with 8% mouse serum (Sigma) for 20 min and added to the BMDMs at a multiplicity of infection (MOI) of 10. Infection was synchronized by centrifugation at 110 × *g* for 5 min. Phagocytosis was allowed to occur for 30 min at 37°C with 5% CO_2_. Infected BMDMs were washed three times with PBS and either collected (0-h time point [*T*_0_]) or incubated in medium with cefotaxime (100 μg/mL; 100× MIC) for 24 h (*T*_24_) at 37°C with 5% CO_2_. For sample collection, BMDMs were washed three times in PBS and lysed with 0.1% Triton X-100 (Sigma) in PBS. Pelleted bacteria were resuspended and diluted in fresh PBS. Bacterial dilutions were plated on agar plates and incubated at 37°C for 16 h. CFU counts were determined and normalized by dividing the number of bacteria at 24 h by the number of bacteria at *T*_0_ for each strain. The obtained values were normalized to those of the respective reference WT strains (14028s or SL1344).

### Fluorescence dilution method *in vivo*.

Salmonella strains carrying the fluorescence dilution plasmid pFCcGi were inoculated in LB medium supplemented with 0.2% l-arabinose and incubated overnight. BMDMs were infected with the preinduced Salmonella as described above and incubated in medium with gentamicin (50 μg/mL). For BMDMs infected with Salmonella SL1344, the medium was supplemented with 2 mM histidine. After 16 h of infection, BMDMs were washed three times in PBS and lysed with 0.1% Triton X-100 in PBS. Pelleted bacteria were resuspended in PBS and subsequently subjected to flow cytometry analysis using a BD LSR II flow cytometer. GFP and mCherry fluorophores were excited at 488 nm and 561 nm, respectively, and detected at 525/50 nm and 615/20 nm, respectively. A total of 10,000 events were recorded for each sample. Data were analyzed with the FlowJo software. Nongrowing bacterial cells were identified and quantified as described above.

### Data availability.

FCS files from single-cell analysis were deposited in the FlowRepository database and can be accessed via https://flowrepository.org/experiments/5329.
