# Supplementary material for: The RNA-Binding Protein ProQ Promotes Antibiotic Persistence in Salmonella
Source: mBio. 2022 Nov 21;13(6):e02891-22. doi: 10.1128/mbio.02891-22 (PMC9765298; doi:10.1128/mbio.02891-22)
Supplement: TABLE S1 [file mbio.02891-22-s0003.docx]

| Strain | MIC (µg/ml) | | |
| --- | --- | --- | --- |
|  | Ciprofloxacin | Cefotaxime | Ampicillin |
| SL1344 wild-type | 0.016 ± 0 | 0.074 ± 0,017 | 0.75 ± 0 |
| SL1344 ∆*proQ* | 0.013 ± 0,002 | 0.084 ± 0,017 | 0.92 ± 0,14 |
| 14028 wild-type |  | 0.064 ± 0 |  |
| 14028 ∆*proQ* |  | 0.064 ± 0 |  |
