# Supplementary material for: The RNA-Binding Protein ProQ Promotes Antibiotic Persistence in Salmonella
Source: mBio. 2022 Nov 21;13(6):e02891-22. doi: 10.1128/mbio.02891-22 (PMC9765298; doi:10.1128/mbio.02891-22)
Supplement: TABLE S5 [file mbio.02891-22-s0005.docx]

| **Genetic background** | **CFU/ml 30 min after infection** | **CFU/ml 24 h after cefotaxime addition** |
| --- | --- | --- |
| SL1344 | 6.3x10^5^ | 612 |
| SL1344 ∆*proQ* | 7.9x10^5^ | 291 |
| 14028 | 1.1x10^6^ | 250 |
| 14028 ∆*proQ* | 1.8x10^6^ | 54 |
