# Supplementary material for: The RNA-Binding Protein ProQ Promotes Antibiotic Persistence in Salmonella
Source: mBio. 2022 Nov 21;13(6):e02891-22. doi: 10.1128/mbio.02891-22 (PMC9765298; doi:10.1128/mbio.02891-22)
Supplement: TABLE S2 [file mbio.02891-22-s0006.docx]

| **Name** | **Relevant details** | **Reference** |
| --- | --- | --- |
| JVS-1574 | *S.* Typhimurium SL1344, *Str^R^ hisG rpsL xyl,* wild-type | (Stocker *et al*, 1983) |
| JVS-11364 | SL1344 ∆*proQ::KanR* | (Smirnov *et al*, 2016) |
| JVS-11365 | SL1344 ∆*proQ* | (Smirnov *et al*, 2016) |
| EHS-2093 | SL1344 ∆*flhDC* | This study |
| EHS-2154 | SL1344 ∆*flhDC ∆proQ* | This study |
| EHS-2391 | SL1344 ∆STM1553::*KanR* | This study |
| EHS-2392 | SL1344 ∆STM1553::*CamR* | This study |
| EHS-2404 | SL1344 ∆*proQ* ∆STM1553::*KanR* | This study |
| EHS-2403 | SL1344 ∆*proQ* ∆STM1553:*CamR* | This study |
| EHS-3284 | SL1344 ∆*flhDC* ∆STM1553::*KanR* | This study |
| EHS-3285 | SL1344 ∆*flhDC* ∆STM1553:*CamR* | This study |
| EHS-3286 | SL1344 ∆*flhDC ∆proQ* ∆STM1553::*KanR* | This study |
| EHS-3287 | SL1344 ∆*flhDC ∆proQ* ∆STM1553:*CamR* | This study |
| 1867, McClelland collection | *S.* Typhimurium 14028 ∆*slyA::KanR* | (Porwollik *et al*, 2014) |
| EHS-1876 | SL1344 ∆*slyA::KanR* | This study |
| EHS-1880 | SL1344 ∆*slyA* | This study |
| EHS-1882 | SL1344 ∆*slyA ∆proQ* | This study |
| EHS-3456 | SL1344 ∆*slyA* ∆STM1553::*KanR* | This study |
| EHS-3459 | SL1344 ∆*slyA ∆proQ* ∆STM1553:*CamR* | This study |
| EHS-2209 | *S.* Typhimurium 14028 wild-type | ATCC |
| EHS-2213 | *S.* Typhimurium 14028 ∆*proQ* | This study |
| SK3313 | *S.* Typhimurium 14028 STM1553::terCATter | (Stårsta *et al*, 2020) |
| SK3318 | *S.* Typhimurium 14028 STM1553::*KanR* | (Stårsta *et al*, 2020) |

Porwollik S, Santiviago CA, Cheng P, Long F, Desai P, Fredlund J, Srikumar S, Silva CA, Chu W, Chen X, Canals R, Reynolds MM, Bogomolnaya L, Shields C, Cui P, Guo J, Zheng Y, Endicott-Yazdani T, Yang HJ, Maple A, Ragoza Y, Blondel CJ, Valenzuela C, Andrews-Polymenis H & McClelland M. (2014) Defined single-gene and multi-gene deletion mutant collections in Salmonella enterica sv Typhimurium. *PLoS One* 9(7):e99820

Smirnov A, Förstner KU, Holmqvist E, Otto A, Günster R, Becher D, Reinhardt R & Vogel J (2016) Grad-seq guides the discovery of ProQ as a major small RNA-binding protein. *Proc Natl Acad Sci U S A* 113(41):11591-11596

Stårsta M, Hammarlof DL, Waneskog M, Schlegel S, Xu F, Gynnå AH, Borg M, Herschend S & Koskiniemi S (2020) RHS-elements function as type II toxinantitoxin modules that regulate intramacrophage replication of Salmonella Typhimurium. *PLoS Genet* 16(2):e1008607.

Stocker BA, Hoiseth SK & Smith BP (1983) Aromatic-dependent ‘Salmonella sp.’ as live vaccine in mice and calves. *Dev Biol Stand* 53:47-54.
