# Supplementary material for: The RNA-Binding Protein ProQ Promotes Antibiotic Persistence in Salmonella
Source: mBio. 2022 Nov 21;13(6):e02891-22. doi: 10.1128/mbio.02891-22 (PMC9765298; doi:10.1128/mbio.02891-22)
Supplement: TABLE S3 [file mbio.02891-22-s0007.docx]

| **Name** | **Details** | **Parental plasmid** | **Reference** |
| --- | --- | --- | --- |
| pAR007 | Empty vector; contains the PLlacO-C promoter and LacI repressor; confers TetR | - | (Rizvanovic *et al*, 2021) |
| pAR009 | Contains IPTG-inducible ProQ; confers TetR | pAR007 | (Rizvanovic *et al*, 2021) |
| pFCcGi | Contains arabinose-inducible GFP and constitutively expressed mCherry; confers AmpR | - | (Figueira *et al*, 2013) |
| pKD4 | Contains an FRT-flanked *KanR* gene | - | (Datsenko & Wanner, 2000) |
| pCP20 | Expresses Flp recombinase for removal of antibiotic resistance cassette; confers CamR | - | (Cherepanov & Wackernagel, 1995) |

Cherepanov PP & Wackernagel W (1995) Gene disruption in Escherichia coli: TcR and KmR cassettes with the option of Flp-catalyzed excision of the antibiotic-resistance determinant. *Gene* 158(1):9–14

Datsenko KA & Wanner BL (2000) One-step inactivation of chromosomal genes in Escherichia coli K-12 using PCR products. *Proc Natl Acad Sci U S A* 97(12):6640-5

Figueira R, Watson KG, Holden DW & Helaine S (2013) Identification of Salmonella pathogenicity island-2 type III secretion system effectors involved in intramacrophage replication of S. enterica serovar typhimurium: Implications for rational vaccine design. *mBio* 4(2):1–10

Rizvanovic A, Kjellin J, Söderbom F & Holmqvist E (2021) Saturation mutagenesis charts the functional landscape of Salmonella ProQ and reveals a gene regulatory function of its C-terminal domain. *Nucleic Acids Res* 49(17):9992–10006
