# Supplementary material for: The RNA-Binding Protein ProQ Promotes Antibiotic Persistence in Salmonella
Source: mBio. 2022 Nov 21;13(6):e02891-22. doi: 10.1128/mbio.02891-22 (PMC9765298; doi:10.1128/mbio.02891-22)
Supplement: TABLE S4 [file mbio.02891-22-s0004.docx]

| **Strain construction** | | |
| --- | --- | --- |
| **Name** | **Sequence (5’-3’)** | **Comment** |
| EHO-1037 | gccgaaattgtgctgcctgtc | Fwd primer to confirm strain constructions in *proQ* |
| EHO-1038 | gcctgctaatgcaagcaggc | Rev primer to confirm strain constructions in *proQ* |
| EHO-1118 | cgttatacgtcacaatgtccataatgtctgagcgctgctagtgtaggctggagctgcttc | Fwd primer for knock out of *flhDC* |
| EHO-1119 | aagttcccaccgcagcagttacagctcgacaattcaagcacatatgaatatcctccttag | Rev primer for knock out of *flhDC* |
| EHO-1120 | tgtcacgaagctgacgagtaga | Fwd primer to confirm strain constructions in *flhDC* |
| EHO-1121 | aatatcggcagcatctcggg | Rev primer to confirm strain constructions in *flhDC* |
| EHO-1574 | gagcagaacgccattcagcc | Fwd primer to confirm strain constructions in STM1553 |
| EHO-1575 | gacatacacgaatccagccgc | Rev primer to confirm strain constructions in STM1553 |
